# Engineering Scalable Manufacturing of High-Quality Stem Cell-Derived Cardiomyocytes for Cardiac Tissue Repair

**DOI:** 10.3389/fmed.2018.00110

**Published:** 2018-04-24

**Authors:** Kaitlin K. Dunn, Sean P. Palecek

**Affiliations:** University of Wisconsin-Madison, Chemical and Biological Engineering, Madison, WI, United States

**Keywords:** cardiomyocyte, maturation, cell manufacturing, human pluripotent stem cells, coculture, cardiac repair, differentiation, regenerative medicine

## Abstract

Recent advances in the differentiation and production of human pluripotent stem cell (hPSC)-derived cardiomyocytes (CMs) have stimulated development of strategies to use these cells in human cardiac regenerative therapies. A prerequisite for clinical trials and translational implementation of hPSC-derived CMs is the ability to manufacture safe and potent cells on the scale needed to replace cells lost during heart disease. Current differentiation protocols generate fetal-like CMs that exhibit proarrhythmogenic potential. Sufficient maturation of these hPSC-derived CMs has yet to be achieved to allow these cells to be used as a regenerative medicine therapy. Insights into the native cardiac environment during heart development may enable engineering of strategies that guide hPSC-derived CMs to mature. Specifically, considerations must be made in regard to developing methods to incorporate the native intercellular interactions and biomechanical cues into hPSC-derived CM production that are conducive to scale-up.

## Structural and Functional Considerations for Cardiac Tissue Regeneration

The heart is a complex organ composed of three layers: the epicardium, myocardium, and endocardium. Within these layers reside many different cell types including cardiomyocytes (CMs), endothelial cells (ECs), smooth muscle cells (SMCs), epicardial cells, fibroblasts, neurons, and immune cells (1). CMs are the cardiac muscle cells, which provide the mechanical contractile function in the heart and reside specifically in the myocardium. They make up only 25–35% of the cells found in the heart (2). There are distinct CM subtypes, including nodal, ventricular, and atrial CMs, which differentially express over 6,274 genes (3). These CM subtypes originate from different mesodermal subtype populations and reside in different locations—ventricular CMs in the ventricles, nodal CMs in the sinoatrial node, and atrial CMs in the atria (4). Additionally, the left ventricle pumps blood throughout the body whereas the right ventricle to the lungs. Thus, the left ventricular CMs must produce higher forces of contraction and require greater oxygen and nutrient uptake. Conversely, atrial CMs require less force generation to pump blood from the atria into the ventricles. The contraction of the heart is controlled by the cardiac pacemaker, which is comprised of sinoatrial node CMs. These nodal CMs exhibit distinct electrophysiological and Ca^2+^ handling properties relating to their primarily stimulatory role (5). Thus, the unique functions of these CM subtypes are not interchangeable.

Throughout development and for normal function, CMs interact with other cell types in the heart. The epicardial cells, cells that comprise the outer layer of the heart, undergo epithelial-to-mesenchymal transition both during heart development and repair to produce SMCs, fibroblasts, and possibly ECs (6, 7). These SMCs, fibroblasts, and ECs interact with CMs in the myocardium to influence their survival and function. The fibroblasts compromise approximately 20% of the non-myocytes found in the heart and are primarily responsible for the extracellular matrix (ECM) deposition in the heart (2, 8). SMCs aid in the regulation of blood flow in the heart. ECs are the most abundant non-myocyte cell in the heart, comprising 60% of the non-myocytes (2). They line the vasculature and aid in the delivery of nutrients and removal of waste. Endocardial ECs specifically line the heart chambers and myocardial ECs comprise the capillaries that directly interact with CMs. Interactions between these cardiac cell types are necessary to support the contractile function of the heart.

Cardiovascular disease is the leading cause of death globally. In 2015, it contributed to the death of about 17.7 million people, which accounts for 31% of the total deaths that year (9). This high mortality rate is caused by the death of millions of CMs, a cell type that has a very low ability to regenerate to replace damaged areas with healthy cells (10). Valvular heart disease and cardiac hypertension slowly kill CMs over time (11). In comparison, myocardial infarctions can cause 25% of the CMs in the left ventricle to undergo cell death in just a few hours (11). During an acute myocardial infarction, a blockage occurs in the blood flow of a coronary artery preventing the delivery of oxygen and nutrients to the cardiac tissue. The CMs in the left ventricle are most impacted by heart attacks due to their high demand of oxygen and nutrients. During the heart’s chronic response to a myocardial infarction, fibroblasts proliferate and form scar tissue, stiffening the heart wall and disrupting the native conduction system, thereby contributing to the likelihood of cardiac failure.

Currently, the only method to completely restore cardiac function for extended duration in patients with advanced cardiac disease is a heart transplant. Alternatively, left ventricular assist devices can temporarily aid the ability of the heart to function but these devices pose significant risks for infection and thrombosis (12). Many efforts are being investigated to repair the damaged cardiac tissue, including creating new heart tissue from stem or progenitor cells or from reprogrammed somatic cells. Some of the most promising stem cell sources for cardiac tissue include both human embryonic stem cells (hESCs) and induced pluripotent stem cells (iPSCs). Other potential cell types that could be used to repair cardiac tissue include the proliferation of a very rare population of adult cardiac progenitor cells (CPCs) or epicardial cells (10). The potential of epicardial cells to form CMs *in vitro* or *in vivo* remains controversial, but they contribute to non-myocyte cell populations in the heart. Also further investigation will be required into methods to stimulate differentiation of adult CPCs, which have very low rates of CM formation, to realize their cardiac regenerative potential (10). The main advantage of using stem cells is that they can be expanded prior to differentiation. Estimates of one billion CMs are required for repair of the ventricle after a myocardial infarction (13). Unfortunately, human pluripotent stem cell (hPSC)-derived CMs are immature, exhibiting the structure and function of developing CMs found in a fetus instead of those in an adult heart (14). On the other hand, reprogramming fibroblasts is a relatively new and still inefficient method, requiring further characterization of the resulting CMs to determine their subtype and maturity (15). For these reasons, most research has focused on using hPSC-derived CMs to replace native CMs cells lost in cardiac diseases.

## Differentiation of hPSCs to CMs

### Generation of Immature hPSC-Derived CMs

**Figure 1:**
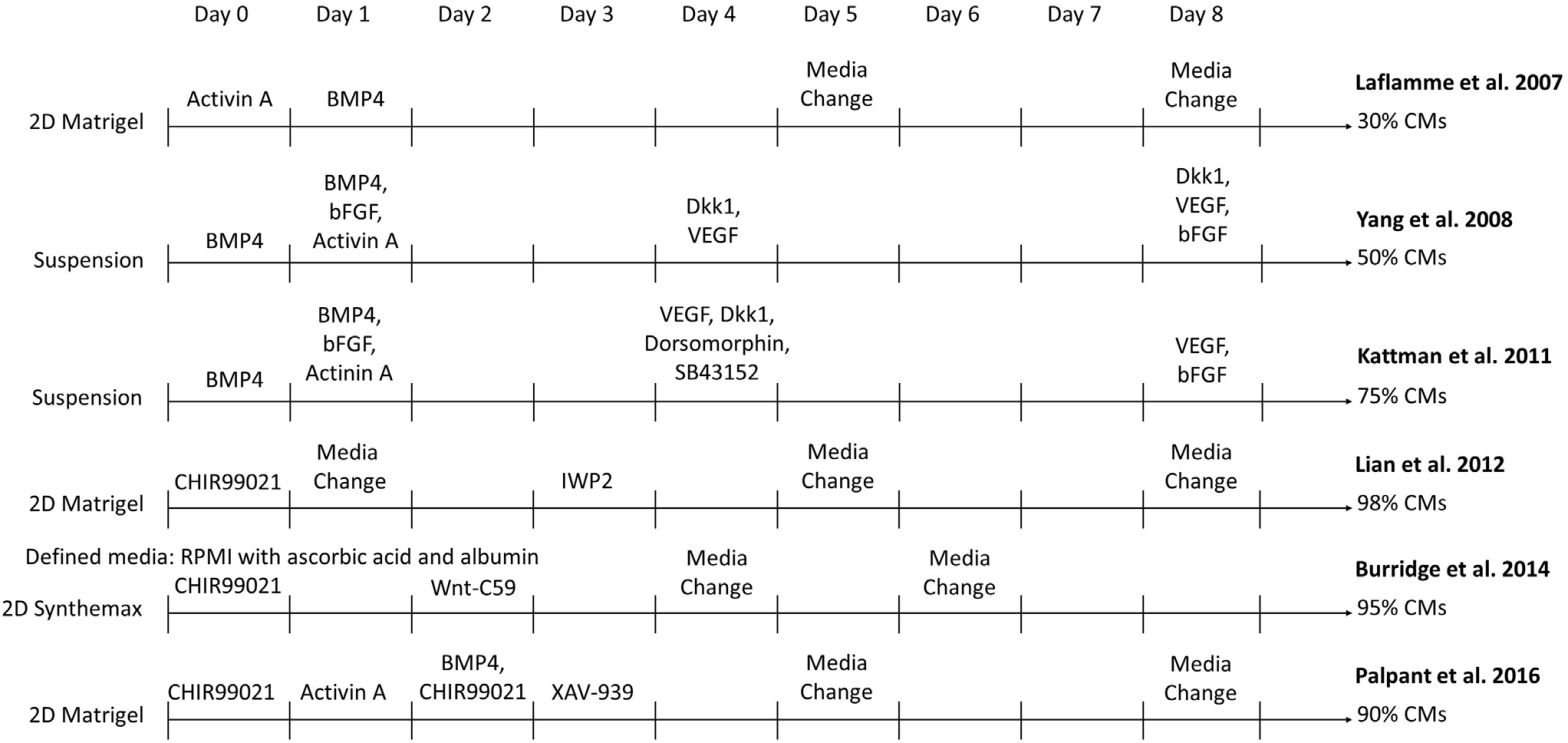
Comparison of select directed differentiation protocols for differentiating human pluripotent stem cells to cardiomyocytes (CMs).

Methods have greatly improved to manufacture sufficient quantities of essentially pure CMs from hPSCs under defined conditions to enable development of cardiac translational therapies. The original differentiation methods relied on isolating small populations of CMs, typically 1–5% of cells, which spontaneously formed in embryoid bodies (EBs) (16, 17). While these initial demonstrations of CM differentiation generated cells for research purposes, advances in yield and purity were necessary to generate enough CMs for investigation of their therapeutic potential. Over the past decade, CM differentiation processes have evolved and become more efficient. Major advances to this method have allowed the differentiation to be optimized, including the determination of pathways that are modulated during CM formation in the embryo, the timing at which to induce these pathway changes, and the ability to activate these pathways in the cells with growth factors and small molecules as seen in Figure 1. In 2007, Laflamme et al. cultured hESCs in a tissue culture plate coated with Matrigel (18). They obtained purities of ~30% CMs through modulation of TGFβ superfamily signaling using Activin A and BMP4 to induce cardiac mesoderm formation (18). In a suspension culture, addition of BMP4, bFGF, Activin A, Dkk1, and VEGF at different stages of differentiation yielded >50% CMs (19). This method was further improved by the inclusion of dorsomorphin and SC43152 (20). In another 2D differentiation approach, Lian et al. generated 80–98% pure populations of CMs solely by modulating the Wnt pathway with the small molecules CHIR99021 and IWP2 (21, 22). Combinations of these strategies incorporated activation of the BMP pathway along with the Wnt pathway modulation to yield ~90% CMs (23). Xeno-free differentiation platforms have been developed by adding ascorbic acid and replacing the B27 supplement with human recombinant albumin or removing the B27 supplement altogether (24, 25). These fully defined, xeno-free methods reduce the variability in media components and eliminate possible patient immune reactions to animal components in the CM product. These protocols can serve as templates to enable the production of CMs at a scale required for regenerative medicines.

### Immature Phenotypes of hPSC-Derived CMs

The lack of mature, adult-like phenotypes in hPSC-derived CMs is a crucial limitation in advancing these cells toward clinical therapies. Their fetal-like state has been linked to arrhythmias after transplantation in large animal models (13). Chong et al. implanted hESC-derived CMs into infarcted macaque hearts through an intramyocardial injection. The immune-suppressed macaques that received the injection experienced irregular heart rates, with premature beating and tachycardia in the ventricle, with one monkey experiencing as many as a thousand non-sustained ventricular tachycardia episodes in a day. Shiba et al. injected CMs differentiated from MHC-matched, allogeneic, monkey induced pluripotent stem cells into infarcted hearts of Filipino cynomolgus monkeys (26). Though the grafts were not rejected and the CMs were able to integrate into the myocardial tissue partially restoring the heart, all the monkeys receiving CMs also experienced ventricular tachycardia episodes for up to 24 h per day. In both studies, the arrhythmias decreased in frequency over time, perhaps due to a degree of *in vivo* maturation. For cell safety and efficacy, these hPSC-derived CMs must be matured enough to significantly reduce the potential to induce arrhythmias upon transplantation.

**Figure 2:**
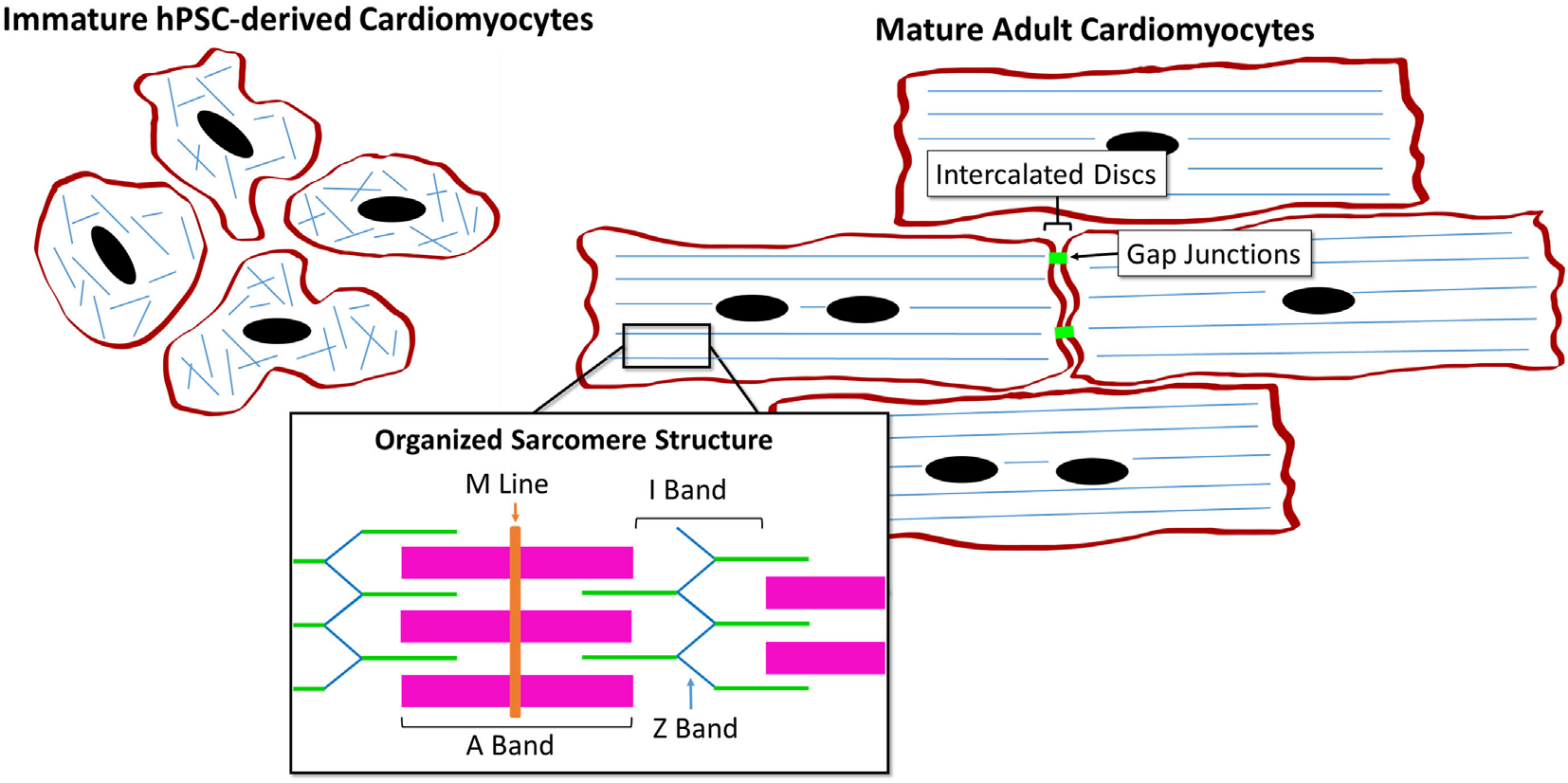
Comparison of human pluripotent stem cell (hPSC)-derived CMs and adult CMs demonstrating the structural and organizational changes during maturation.

The hPSC-derived CM immature phenotype is characterized by a difference in marker expression, electrical and mechanical functionality, metabolism, calcium handling, and morphology in comparison to adult CMs, as summarized in Table 1. Structurally, hPSC-derived CMs are smaller, rounded cells, which more closely resemble embryonic CMs (27). In comparison, adult CMs have a much more elongated, rod-like shape as seen in Figure 2 (28). Around 30% of adult CMs are multinucleated (29). Additionally, major changes affecting CM contractility occur in the organization of the CM sarcomeres and myofibrils during maturation (30). The anisotropic alignment of adult CMs is important to allow efficient propagation of electrical signals (31, 32). These are aided by the formation of connexin-43 (Cx43)-containing gap junctions between the cells (33).

**Table 1:** Comparison of human pluripotent stem cell (hPSC)-derived cardiomyocytes (CMs) and adult CMs to demonstrate the changes during maturation.

Differences between hPSC-derived CMs and adult CMs		

	hPSC-derived CMs	Adult CMs
**Cell structure and organization**		
Cell shape	Round	Rod-like
	Mono-nucleated	30% multinucleated
Cell alignment	Disordered	Anisotropic alignment
Sarcomere structure	Disordered sarcomere	I bands, M lines, A bands, Z bands, and intercalated disks
Sarcomeric gene and protein expression	Low expression	High expression of *MYL2, TNNI3, ACTN2, MYH7, MYL3, TNNC1, TNNT2*
	MLC-2a	MLC-2v (ventricular CMs)
	α-MHC	β-MHC
	ssTnI	cTnI

**Electrophysiology**		
Upstroke velocity	2 to >200 V/s	300 V/s
Resting membrane potential	−58 mV	−80 mV
Ion channel gene expression	Low expression	High expression of *CACNA1C, HCN4, SCN5A, ATP2A2, KCND3, and KCNH2*

**Contractility**		
Excitation–contraction coupling	Low coupling, spontaneous beating	High coupling, quiescent
Contraction force	~30 nN	In the order of micronewtons
Gap junctions	Low expression	High expression, including connexin-43

**Ca^2+^ handling**		
T-tubules	Not present	Present
Conduction velocity	2.1–20 cm/s	41–84 cm/s
Metabolism	Glucose oxidation	Fatty acid β-oxidation

Many CM genes are more highly expressed in adult CMs than in hPSC-derived CMs. These genes encode ion channels, calcium regulators, sarcoplasmic reticulum transporters, and sarcomeric proteins including, but not limited to: *CACNA1C, HCN4, SCN5A, ATP2A2, MYL2, TNNI3, ACTN2, MYH7, MYL3, TNNC1, TNNT2, KCND3*, and *KCNH2* (34). Expression of different isoforms of sarcomeric proteins switch during CM maturation. Immature CMs express the slow skeletal isoform of troponin I (*TNNI1*) while more mature cells express the cardiac isoform (*TNNI3*) (35). Ventricular CMs primarily express MLC-2a and α-MHC early in development but upregulate MLC-2v and β-MHC as they mature (36). hPSC-derived CMs spontaneously beat while adult ventricular CMs are quiescent, requiring pacing by the nodal CMs (37). Also, the primary mode of carbon metabolism of CMs changes from glucose oxidation to fatty acid β-oxidation during development (38). The force of the adult CM contraction is on the order of micronewtons, much larger than the reported hPSC-derived CM force of ~30 nN (39, 40).

hPSC-derived CMs have very immature, irregular electrophysiological responses. Their upstroke velocity ranges from 2 to greater than 200 V/s in comparison to 300 V/s in adult CMs (41). The immature CMs have reduced excitation–contraction coupling and a higher resting membrane potential of −58 mV, compared to the adult CM resting membrane potential of –80 mV (37). hPSC-derived CMs lack T-tubules, which aid in rapid signal transmission between cells through the sarcoplasmic reticulum (28). Instead, hPSC-derived CMs rely on trans-sarcolemmal calcium influx (28), which results in a reduced conduction velocity of 2.1–20 cm/s compared to 41–84 cm/s in adult CMs (42–44).

One strategy to induce maturation of hPSC-derived cells involves implanting immature cells *in vivo*. This method has proved effective in maturing other hPSC-derived cell types including neural stem cells and pancreatic beta cell progenitors. For example, hESC-derived neural stem cells were implanted into C5 spinal cord lesion sites and increasing numbers of cells producing NeuN, a mature neural marker, were found throughout the following year (45). In addition, hESC-derived pancreatic progenitors differentiated into mature insulin-producing β-cells that expressed prohormone convertase enzymes upon implantation below the left kidney of immune-deficient mice (46, 47). Indeed, there is evidence that hPSC-CMs undergo a degree of maturation after implantation to the heart. For example, Kadota et al. demonstrated that implantation of CPCs and CMs into adult rat hearts exhibited maturation over time, as assessed by CM cell size, sarcomere length, and cTnI expression (48). However, after 3 months these cells had not yet reached the size of the rat CMs, suggesting they were still relatively immature. Some maturation was also seen over time in hESC-derived CMs grafts that were implanted in the macaques after they underwent an induced myocardial infarction, though many of the cells in the center of the grafts remained immature (13). The transplanted CMs in the graft core were not fully aligned, displayed low α-actinin expression and were much smaller than the hESC-derived CMs at the edge of the graft. Even if it were effective, implantation of hESC-CMs in an animal does not represent a realistic approach to scaling manufacturing of cells for human therapeutics. Therefore, other methods must be pursued to mature hPSC-derived CMs in order to improve their safety and efficacy.

### Design Considerations to Induce hPSC-Derived CM Maturation

A tradeoff between functional maturity and engraftment efficiency complicates selection of an ideal maturation state for transplanting hPSC-CMs. Funakoshi et al. reported that immature day 20 iPSC-derived CMs injected intramyocardially into mouse hearts engrafted to a greater extent than more mature day 30 CMs, based on the number of human CMs found throughout the heart 2 months after transplantation (49). Testing on large animal models with a more similar physiology to human hearts will need to be done to determine the level of maturation that would be optimal for both integration and functional improvements in developing human cell-based therapies. Toward the goal of developing transplantable human iPSC-derived organs, Wu et al. are developing human–pig chimeras by incorporating human iPSCs into the inner cell mass of a pig blastocyst (50). Additionally, standardized maturity metrics are needed to compare how different signals and environments affect hPSC-CM maturation. Bedada et al. profiled the switch in expression of ssTnI to the cardiac isoform cTnI through cardiac development (35). Mouse stem cell-derived and rodent neonatal CMs exhibit significant levels of cTnI but human iPSC-derived CMs predominantly expressed ssTnI even after 9.5 months in culture. They suggested that the ratio of cTnI:ssTnI may serve as a useful marker for later stages of hPSC-derived CM maturation (35). However, the relationship between cardiac gene isoform switching and electromechanical and metabolic phenotypes has not yet been established. While determining the extent of maturation that leads to optimal regenerative performance and setting benchmarks to define when this level has been reached will be important steps toward creating cardiac cell-based therapies, a significant number of studies have been performed to attempt to accelerate maturation of hPSC-derived CMs through both biochemical and biophysical methods.

Several strategies to enhance maturation of hPSC-derived CMs have been described in recent years, with limited success in terms of rate and extent of maturation achieved. Both Ivashchenko et al. and Lundy et al. characterized the temporal changes in iPSC-derived CM maturation throughout time in culture, up to 80 and 120 days, respectively (37, 51). Though the cells increased in size, organization, sarcomere length, expression of key cardiac genes, responsiveness to ion channel activators and inhibitors, and electrophysiology, they were still immature compared to adult CMs. Even though extended culture can be an effective strategy to mature hPSC-derived CMs, the amount of time required is generally not compatible with manufacturing timelines. Strategies to accelerate the rate of maturation include mechanical stimulation, electrical stimulation, altering ECM composition and substrate stiffness, directing cellular alignment, and coculture of the CMs with the other cell types prominent in the heart. These strategies provide differentiating CMs with cues found in the developing heart environment and their ability to induce maturation will be discussed in detail in Section “Incorporation of Acellular Methods to Induce hPSC-Derived CM Maturation.”

### The Impact of Non-Myocytes on hPSC-Derived CM Maturation

In a developing heart the CMs are in direct contact with and receive soluble cues from a variety of other cell types including fibroblasts, SMCs, ECs, and epicardial cells. In fact, when these interactions are eliminated in mouse embryos, the heart is unable to form correctly. For example, when Luxán et al. specifically inactivated Delta-Notch pathway components *Mib1* or *Jag1* in mouse myocardium or *Notch1* in the endocardium, the resulting hearts demonstrated left ventricular non-compaction cardiomyopathy (52). Similarly, Lavine et al. found *Fgf9* upregulation in both mouse endocardium and epicardium (53). *Fgf9* knockout resulted in decreased CM proliferation and dilated cardiomyopathy, a result that was also achieved by knocking out myocardium-specific expression of the receptors *Fgfr1* and *Fgfr2*.

As *in vitro* CM differentiation processes have evolved to become more efficient, signals from other cell types in a more heterogeneous population have been lost, perhaps altering the ability of the CMs to achieve mature phenotypes. For example, Kim et al. purified hESC-derived CMs from EBs at different time points and further cultured the cells to 60 days (54). The CMs maintained in culture with non-CMs for longer time displayed enhanced maturation, including elevated expression of cardiac ion channels, electrophysiological maturity, and responsiveness to HCN, Na^+^, and Ca^2+^ ion channel blockers, compared to CMs purified earlier. It is not clear why CMs cultured with non-CMs for longer time achieved greater maturation than the CMs in monoculture. EBs are known to contain many cells types in addition to CMs, including endodermal, ECs, neural crest, and epicardial cells (53). With <7% of the EB composition being CMs, this study suggests that non-myocytes may play an important role in phenotypic maturation of hPSC-derived CMs.

**Figure 3:**
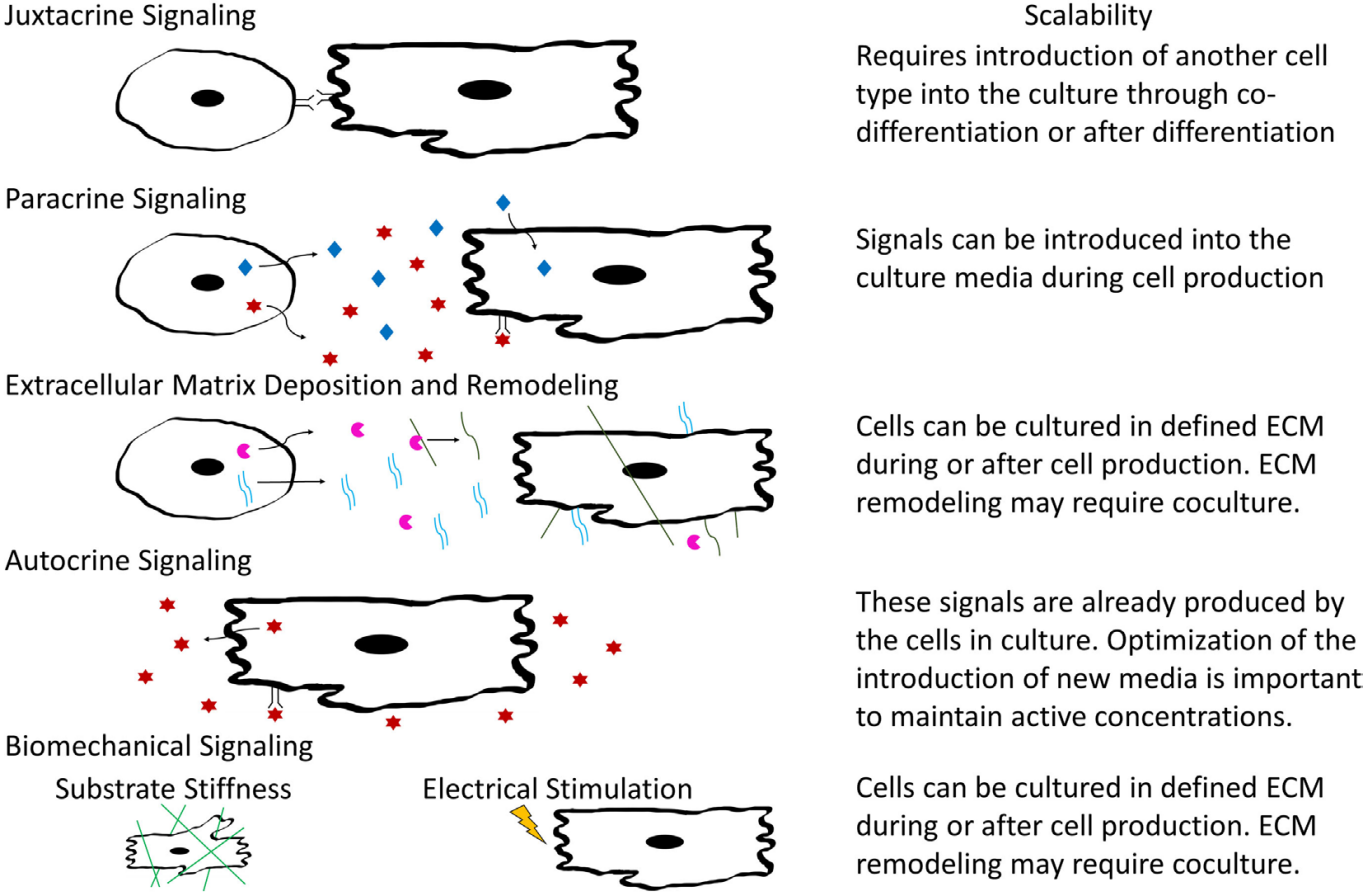
Schematic illustrating types of intercellular interactions and their scalability for inclusion into large-scaling manufacturing.

In tissue development and maintenance, cells interact in a variety of manners including autocrine and paracrine signaling, juxtacrine and biomechanical cues, and through remodeling of ECM components as shown in Figure 3. Identifying how various cardiac cell types impact CM phenotypes will be important for designing appropriate coculture systems that stimulate maturation of hPSC-derived CMs in a manufacturing setting. Some cues, such as soluble factors, are amenable to scale-up, while others such as electrical and mechanical signals are more complicated to integrate into a bioreactor. The remainder of this review will focus on our current understanding of the role of both intercellular interactions and acellular methods to induce maturation in hPSC-derived CMs and cardiac tissues and discuss the logistics of incorporating these interactions into scalable CM manufacturing processes.

## Mimicking Intercellular Interactions *Via* Soluble Factors and ECM

**Figure 4:**
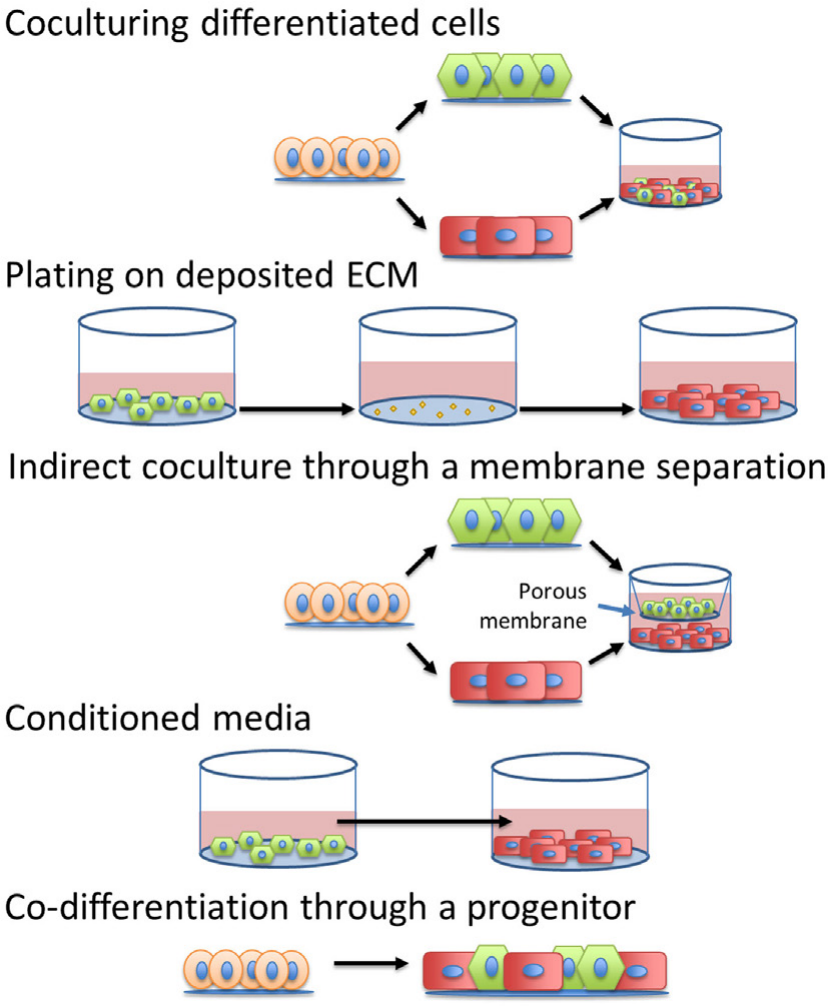
Different strategies to introduce intercellular interactions during human pluripotent stem cell-derived cardiomyocyte manufacturing.

The simplest method to incorporate intercellular signals into the production of CMs would be through the addition of soluble factors into the differentiation platform. If the pathways or molecules through which various cardiac cells interact with CMs to accelerate maturation were identified, then these signals or other molecular modulators of these pathways could be introduced into the culture at specific times by manipulating medium composition. Additionally, identification of the defined, cardiac tissue inspired ECM for hPSC-derived CM maturation could mitigate the need to integrate other cell types into the production hPSC-derived CMs. A summary of the methods to induce maturation, shown in Figure 4, and their effects on specific CM phenotypes can be seen in Table 2.

**Table 2:** Summary of improvements to maturation phenotypes through different cues.

Methods to induce human pluripotent stem cell-derived cardiomyocyte maturation										
	Cell shape	Cell alignment	Gap junctions	Sarcomere structure	Sacromeric gene or protein expression	cTnI:ssTnI ratio	Electrophysiology	Ion channel gene or protein expression	Contractility	Ca^2+^ handling
**Cell-secreted factors**										
Fibroblast-conditioned media (56)	+		0				-	-		
Indirect fibroblast coculture (56)							+			
EC-lysates (61)				+						
EC-conditioned media (61)				0						
EC-conditioned media (62)									0	0
**Juxtacrine**										
Direct fibroblast coculture on fibroblast extracellular matrix (ECM) in comparison to indirect coculture (58)					+					
Direct EC coculture (61)	+	+		+	+					+
Direct EC coculture (62)									+	+
Fibroblast and EC coculture (78)	+	+	+		+			+		+
Fibroblast and EC coculture (79)					+			+	+	+
Direct EC coculture (91)					+	−	0	+		+
**ECM**										
Fibroblast-deposited ECM (58)	+		+		+					
EC-deposited ECM (61)				0						
Decellularized adult bovine heart ECM in comparison to decellularized fetal heart (97)					+			+		+
**Metabolite and hormone**										
Tri-iodo-l-thyronine (14)	+			+	-			+	+	+
Glucocorticoid signaling (64)				+		0			+	+
Galactose and fatty acid carbon source (67)	+	+		+	+	+	+	+	+	+
**Biomechanical**										
Cyclic stretch (92)	+	+			+			+		
Cyclic Stretch with fibroblasts and ECs present (86)			+		+				+	+
Cyclic stretch (93)	+	+	+		+			+		+
Culture on soft PDMS in comparison to glass (98)			+			+		+		+
Culture on aligned fibers (99)		+		+			+		+	+
**Electrical**										
Electrical pacing (94)	+				+		+	+		+
Electrical pacing (95)		+	+	+		+		+	+	+

*+ symbolizes an improvement of maturation, 0 is no significant improvement, − is a decrease in maturation. If left blank, then that type of analysis was not reported*.

### Interactions With Fibroblasts

Fibroblasts are a vital cell type for cardiac function and may be essential for cardiac maturation. They are responsible for secretion of growth factors, ECM deposition and remodeling, and even connect to CMs through connexins to aid in electrical signal propagation (55). Several studies have employed different methods and platforms to simulate and incorporate coculture of different fibroblast populations with either hPSC-derived or neonatal CMs. Culturing rat neonatal CMs in rat neonatal cardiac fibroblast-conditioned medium induced proarrhythmic changes (56). After 24 h in the conditioned medium, the CMs had a prolonged action potential duration and a slower conduction velocity, measured by single-cell electrophysiology, compared to the unconditioned control, suggesting the fibroblast-conditioned medium impeded electrophysiological maturation. This adverse effect was not seen when the CMs were able to interact with the cardiac fibroblasts in a non-contact coculture (56). By contrast, the non-contact coculture appeared to enhance structural maturation of the CMs, increasing CM cell size and expression of β-MHC suggesting that intercellular crosstalk is important in regulating the signals between the fibroblasts and CMs for CM maturation.

The cardiac ECM is important for distributing mechanical forces, conveying biochemical and biomechanical signals, and providing structural integrity to the surrounding tissue (55). Since it is essential to transmitting signals between CMs and the neighboring tissue, the ECM composition likely impacts the ability of hPSC-derived CMs to mature. Thus, it is likely a direct coculture or culture on fibroblast-derived ECM can influence hPSC-derived CM cell states (57). Indeed, Suhaeri et al. developed a scaffold coated with mouse fibroblast-deposited ECM that, when used to culture hESC-derived CMs, caused enhanced maturation as demonstrated by enhanced transcription of *TNNT2*, upregulation of Cx43 and α-actinin expression, and increased cell length-to-width ratio (58). Rat neonatal CMs also exhibited enhanced cardiac gene and protein expression, cell hypertrophy, increased sarcomere length, and more extensive cell multinucleation in both direct contact and non-contact cocultures with fibroblasts while cultured on fibroblast-derived ECM. While the neonatal rat CMs approached adult-like phenotypes with respect to cell shape and electrophysiology, the hESC-derived CMs remained more similar to embryonic CMs in their shape. Minimal differences were seen between the contact and non-contact neonatal CM-fibroblast cocultures on fibroblast-derived ECM (58).

Together these studies demonstrate that a non-contact coculture with fibroblasts and fibroblast-derived ECM can enhance CM maturation and can recapitulate the majority of effects from a direct contact coculture, pointing toward ECM deposition and remodeling along with paracrine secretion being the main methods of interaction between the fibroblasts and CMs for CM maturation. While known paracrine factors could be added to culture media, cell-deposited ECM could be introduced into large-scale production through either direct coculture, co-differentiation, or through pre-depositing ECMs onto the substrate before introducing the CMs into the culture.

No evidence so far indicates the necessity of having direct cell-cell contact between CMs and fibroblasts, though incorporation of fibroblasts directly into culture with CMs would allow the fibroblasts to deposit their ECM and secrete paracrine factors. It is also not yet evident whether cardiac-specific fibroblasts affect CM maturation to a greater degree than fibroblasts harvested from other tissues. Cardiac fibroblasts are largely responsible for synthesizing cardiac ECM components, including collagens I and III which together comprise 91% of the total collagen in the heart (8). The ECM also includes CM-produced collagen IV and other components including collagen V and VI, fibronectin, laminin, elastin, and fibrillin (57). Unlike other fibroblasts, cardiac fibroblasts specifically express DDR2 (57). In addition to other paracrine and juxtacrine interactions, further research should investigate cardiac fibroblasts, in comparison to others found in the body, to determine the specific factors and ECM components they produce to accelerate maturation of hPSC-derived CMs.

### Interactions With ECs

As the most numerous cell type in the myocardium besides CMs, ECs are in close contact with CMs throughout heart development, delivering nutrients and removing wastes *via* the circulatory system (2). EC-derived factors may also regulate development and maturation of CMs. For example, endocardial ECs have been shown to produce neuregulin-1, a paracrine signaling factor that can induce electrophysiological maturation in hPSC-CMs (59, 60). To investigate whether rat arterial ECs could enhance hPSC-derived CM maturation, Lee et al. incorporated either EC lysates, EC-generated ECM, or EC-conditioned medium into the CM culture in addition to direct contact coculture of two cell types (61). Both direct coculture and EC lysates enhanced CM maturation, including better-organized sarcomeres, greater cell elongation and alignment, and improved Ca^2+^ handling, compared to CMs in monoculture. However, EC-conditioned medium and EC-derived ECM had no detectable effect on maturation. Additionally, they found that the EC-induced changes in CM maturity were not replicated by mouse cardiac fibroblast coculture (61). ECs from rat fat, aorta, and heart induced similar effects on CM maturation, suggesting that the EC-derived effects on CM maturation are a general endothelial property. Direct EC coculture and EC lysates induced the CMs to upregulate expression of four specific microRNAs, miR-125b-5p, miR-199a-5p, miR-221, and miR-222. Transfection of these microRNAs into CMs induced a degree of CM maturation, although not to the same extend as direct EC coculture (61). Adding microRNAs or other genetic targets of maturation pathways may be a facile method to simulate the effects of coculture in a CM manufacturing process, although more research is necessary to determine the mechanisms by which CMs sense and respond to cues produced by other cardiac cell types. Further investigation by Pasquier et al. saw improvements in the chronotropy and synchrony of hESC-derived CMs when in direct coculture with E4orf1-transfected human umbilical vein ECs in comparison to both EC-conditioned media and monoculture (62). This study further suggests the importance of juxtacrine signaling between the ECs and CMs for CM maturation. Notably, ECs also may aid in CM survival after transplantation due to their ability to vascularize the tissue and therefore are important to include in cardiac regenerative therapies in addition to possible CM maturation effects.

### Hormone and Metabolite Induction of hPSC-CM Maturation

Alternatively, biochemical activation of cardiac maturation pathways may be an effective strategy for manufacturing more mature CMs. Tri-iodo-l-thyronine (T3), a hormone synthesized by the thyroid, has been shown to decrease fetal gene expression and induce an isoform switch from fetal to adult titin in embryonic rat CMs (63). T3 treatment increased iPSC-derived CM cell size and elongation, increased contractility, and increased sarcomere length after 1 week compared to untreated iPSC-derived CMs when the cells were treated with the compound for a week (14). Interestingly, expression of α-MHC was substantially upregulated following T3 treatment, which may indicate specification to atrial CMs. Kosmidis et al. investigated the ability of glucocorticoid signaling, which is known to enhance maturation of all organs in the fetus, to mature hPSC-derived CMs (64). Treating hESC-derived CMs with the synthetic glucocorticoid dexamethasone increased sarcomere length and force of contraction. A combination of dexamethasone and T3 applied to human iPSC-CMs cultured on a Matrigel substrate induced t-tubule network formation and enhanced excitation-contraction coupling (65). Finally, it may be possible to induce hPSC-derived CM maturation through the metabolites provided in the culture media. Bhute et al. found a substantial shift in the metabolism of the hESC-derived CMs as they aged from 1 to 3 months old *in vitro* (66). Aging in culture significantly upregulated phospholipid metabolism, pantothenate and Coenzyme A metabolism, and fatty acid oxidation and metabolism. It may be possible to induce these changes by altering media formulations. Indeed, Correia et al. found that switching to a medium containing galactose and fatty acids as primary carbon sources, rather than of glucose, forced the hPSC-derived CMs to mature at a faster rate (67). These cells demonstrated enhanced contractility, calcium handling, and a more elongated cell shape than CMs cultured in medium containing glucose. Altogether these studies illustrate the potential of regulating hPSC-derived CM maturation *via* known molecular and metabolic modulators of heart maturation. Addition of galactose and fatty acids along with T3 and dexamethasone could easily be incorporated into large-scale production of hPSC-derived CMs through culture media optimization.

## hPSC-Derived CM Maturation in Microtissues

While the addition of soluble factors or fibroblast-derived ECM may not be sufficient to fully mature hPSC-derived CMs, these strategies represent a step in the right direction. Signaling through direct cell-cell contact is also important for cardiac maturation. Also, incorporating hPSC-derived CMs into a scaffold with other cell types may enhance engraftment and survival *in vivo* (68). For these reasons, cardiac microtissues have been investigated as potential regenerative therapies. To create these microtissues, researchers have combined fibroblasts, SMCs, and ECs with CMs by separately differentiating the cells from stem cells or harvesting them from primary sources, and then constructing the tissue. Initially the strategy to combine multiple cell types into a cardiac microtissue was explored to enhance CM survival and engraftment after transplantation, but effects of intercellular interactions on CM phenotypes were observed in these tissues. Alternatively, it may be possible to use the innate ability of certain CPCs to create a microtissue in which the different cardiac cell types spontaneously organize as they differentiate, which will be discussed in Section “Creating Cardiac Tissues *via* Morphogenesis of CPCs.”

In the past few years, hPSC differentiation protocols have been developed to generate relatively pure populations of multiple cardiac cell types in addition to the CMs described in Section “Generation of Immature hPSC-Derived CMs.” Pure populations of CD34^+^ cells, which can give rise to both ECs and SMCs, are obtainable using either MEK/ERK and BMP4 pathway or Wnt pathway activation, followed by magnetic activated cell sorting (MACS) (69, 70). Lui et al. used VEGF-A to drive the formation of cardiac-specific ECs from Isl1^+^ CPCs (71). Purification of CD31^+^CD144^+^ cells was achieved by FACS with antibodies for both CD31^+^ and CD144^+^ surface markers. Epicardial cells and their derivatives have also been differentiated from hPSCs *via* an Isl1^+^Nkx2-5^+^ progenitor. Iyer et al. utilized the WNT3A, BMP4, and RA pathways to create WT1^+^ epicardial cells whereas Bao et al. generated similar cells by stage-specific modulation of the Wnt pathway (72, 73). The resulting epicardial cells were 80–100% pure and could undergo epithelial-to-mesenchymal transition using TGFβ1 together with PDGF-BB or bFGF to generate SMCs and FGF treatment to create fibroblasts (6, 73). Bao et al. also demonstrated that hPSC-derived epicardial cells have the capacity to differentiate to cells expressing endothelial markers after VEGF treatment, but this process remains inefficient (74). hPSC-derived epicardial cells may be differentiated to epicardial-derived cells and then combined with hPSC-derived CMs to form cardiac tissues, or hPSC-derived epicardial cells may be directly incorporated into the cardiac tissues.

Initial attempts to generate cardiac tissues often utilized primary cells as a proof of concept to demonstrate the benefits of including these cells into microtissues in comparison to a CM-only graft. For example, Stevens et al. found that incorporation of human umbilical vein endothelial cells (HUVECs) and mouse embryonic fibroblasts (MEFs) into spheroids containing hESC-derived CMs greatly enhanced the survival of the CMs after transplantation into nude rat hearts (75). *In vitro*, ECs have the capacity to form tube-like vascular structures, though they are generally unstable and often require specific growth factors and 3D ECM or other scaffolds to form. In the presence of fibroblasts, these vascular-like structures were able to form and were maintained and stabilized without specific growth factor supplementation (76). When MEFs were cocultured with hESC-derived ECs and CMs on Matrigel in poly(lactic-co-glycolic acid) sponges *in vitro*, the stability of the tubes was enhanced and the CMs exhibited increased proliferation and expression of MLC-2v (76). Inclusion of ECs aided CM survival after transplantation of hESC-derived cardiac patches over the anterior cardiac wall of infarcted rat hearts (75, 77). The vascular structures in the patch were able to connect to the host capillaries as shown by the staining of Indian ink that was injected into the inferior vena cava (77) and by the presence of leukocytes and Ter-119-positive red blood cells inside the vessels (75). It is not entirely clear whether the EC-mediated vascularization improved CM survival by enhancing delivery of oxygen and nutrients to the graft, or if paracrine and juxtacrine signaling influenced CM fate.

Additionally, combining multiple different cardiac cell types into cardiac tissue constructs has elicited greater maturation than individual cell types, suggesting additive or synergistic effects. Vuorenpää et al. found that fibroblasts together with ECs helped mature CMs (78). They seeded HUVECs and human foreskin fibroblasts first, allowing the cells to spontaneously form a vascular-like network in the culture dish, before adding iPSC-derived CMs. This caused the resulting CMs to orient longitudinally and to become larger. In a similar experiment, Ravenscoft et al. cultured human primary cardiac fibroblasts and ECs with hESC-derived CMs for 2 weeks (79). The resulting CMs exhibited increased contractile response to drugs targeting the β1-adrenergic receptor, EGFR-1/EGFR-2 receptor, or Na/K^+^ ATPase and the increased expression of *S100A1, TCAP, PDE3A, NOS3*, and *KCND3* in comparison to either a monoculture or the combination of CMs with either ECs or fibroblasts alone. This response to the pharmacological agents was elicited by cardiac-specific fibroblasts and ECs, but not dermal fibroblasts or ECs, further suggesting a unique capacity for cardiac-specific cells in maturing hPSC-derived CMs. Though cardiac fibroblast and EC coculture improved gene expression in the CMs, they were still much more representative of fetal CMs than adult CMs.

Further microtissue design and evaluation should test the ability of hPSC-derived cell types to improve the functionality of hPSC-derived CMs. Production of cardiac tissues containing multiple cell types including ECs, fibroblasts, and possibly SMCs or epicardial cells will need to be investigated and optimized. These cardiac microtissues will likely need additional exogenous stimulation *via* biochemical and/or biophysical cues to achieve sufficient maturation.

## Creating Cardiac Tissues *Via* Morphogenesis of CPCs

Instead of independently differentiating various cardiac cell types then combining them to create a cardiac microtissue, it may be advantageous to start with a CPC that can form the desired cell types and differentiate these progenitors in such way that they form organized cardiac structures. If differentiation can be spatially and temporally controlled, one may be able to manufacture cardiac tissues similar in composition and structure to the native myocardium, incorporating key factors that impact CM maturation and survival upon engraftment.

The adult heart contains rare populations of adult CPCs that can differentiate into CMs, ECs, SMCs, and fibroblasts (80). Different markers have been used to identify these adult CPCs including Sca-1 and c-kit, with consensus still needing to be reached on each populations’ potential to form CMs (80–82). Alternatively, CPCs found during development and differentiation of hPSCs to CMs are characterized primarily by the expression of Nkx2.5, Isl1, Flk-1/KDR, and PdgfR-α (20). These hPSC-derived CPCs are multipotent and can further differentiate to epicardial cells, ECs, SMCs, and CMs *in vitro* (71, 73, 83–85). While these CPCs have the capacity to form myocardial cell types, this potential has not yet fully been harnessed to manufacture cardiac tissues *in vitro*. Ruan et al. utilized an hPSC-derived KDR^+^PDGFRα^+^ progenitor to create cardiac tissue constructs, co-differentiating the CPCs in a medium containing VEGF into CMs, SMCs, and ECs, which organized into vascular structures containing lumens (86). Interestingly, 3D differentiation favored CM generation while tissues differentiated in 2D contained a much greater SMC population. One caveat in using the CPCs for engineering cardiac tissues is that it is difficult to fully control the differentiation, with up to 40% of their constructs composed of unidentified cell types (86). Though use of hPSC-derived CPCs may provide a seemingly facile, development-inspired approach for engineering myocardial tissues, progress must be first made to understand how to expand and control differentiation of these cells *in vitro* to generate sufficient quantities of therapeutically relevant cardiac tissues.

In fact, several recent advances in expanding and differentiating CPCs have opened the possibility of implanting CPCs for cardiac regeneration. Isolating CPCs from cardiac tissue and expand these CPCs *in vitro* is challenging (87). Only recently, Birket et al. discovered that by genetically modifying hESCs to allow doxycline-induced *MYC* expression, the CPC population could be maintained for up to 40+ doublings with the addition of IGF-1 and a hedgehog agonist (88). Though the genetic modification to stimulate *MYC* expression may limit the potential to use these cells in regenerative therapies, they will likely prove beneficial to study mechanisms of self-renewal and differentiation fates. Alternatively, two teams have reported methods to reprogram murine fibroblasts into induced CPCs (iCPCs) that can be expanded *in vitro* (15, 89). Lalit et al. induced expression of the cardiac transcription factors and chromatin regulators *Mesp1, Gata4, Tbx5, Baf60c*, and *Nkx2-5* in the fibroblasts (15). Zhang’s method utilized the small molecules B431542, CHIR99021, parnate, and forskolin together with induced expression of *Oct4* (89, 90). Both methods resulted in Flk-1^+^PdgfR-α^+^ iCPCs which were purified and then expanded in medium containing Wnt and JAK/STAT pathway activators (15) or containing BMP4, Activin A, a Wnt inhibitor, and an inhibitor of FGF, VEGF, and PDGF signaling (89). When transplanted into mouse hearts, these cells exhibited the capacity to differentiate into SMCs, ECs, and CMs, but did not form teratomas. The expandable iCPCs generated tissues comprised of approximately 60% SMCs, 7% ECs, and 30% CMs (89). This propensity to differentiate to SMCs may be a consequence of the fibroblast origin of the iCPCs. Finally, reprogramming of fibroblasts to iCPCs has not yet been demonstrated in human cells and further characterization of the resulting CMs need to be done to determine their subtype specificity and maturity. Therapeutic delivery of reprogrammed iCPCs may eliminate the need to terminally differentiate stem cells to cardiac cell types *in vitro*, but we need a better fundamental understanding of how to control differentiation fates and tissue morphogenesis in order to reliably manufacture structurally organized and functional cardiac tissues from iCPCs.

This concept of co-differentiation was used to direct hESCs to a mixed population of CMs and ECs using culture conditions permissive for differentiation to both cell types (91). Addition of VEGF at the same time as inhibition of Wnt signaling generated a population comprised of ~50% CMs and ~16% cardiac-specific *GATA4*^+^ ECs by day 10 after initiation of differentiation. It is not clear whether the VEGF directed a cardiac progenitor to an endothelial fate or provided a selective growth advantage to ECs in the differentiating culture. The CMs and ECs were purified then recombined to form a cardiac microtissue with enhanced CM maturity in their ion channel gene expression which was upregulated compared to CMs alone. These microtissues formed from co-differentiated CMs and ECs also exhibited increased sensitivity to the Ca^2+^ inhibitor verapamil and the β-adrenoreceptor agonist isoprenaline, signs of functional maturation. By contrast, the microtissues formed from co-differentiated CMs and ECs contained a lower cTnI:ssTnI ratio than the CMs alone, suggesting that co-differentiation did not induce myofilament maturation.

Co-differentiation allows cross talk between developing cell types throughout the differentiation process, similar to what occurs in the embryonic heart during development, while combining cells after differentiation may fail to provide intercellular differentiation and maturation cues during the most impactful developmental stages. However, co-differentiation will likely be more difficult to implement in a manufacturing setting because of challenges in controlling the ratio and organization of multiple cell types and the potential need to purify and recombine cells into tissues if they do not spontaneously assemble into appropriate structures. With enough control of the differentiation and morphogenesis processes, it may be possible to engineer the cells to autonomously form organized cardiac tissue structures, enhancing their function and ability to engraft into an adult heart. Further research will be needed to achieve this level of control through design of effective strategies that permit the formation of structured tissues from mixed populations of differentiating cardiac cells.

## Incorporation of Acellular Methods to Induce hPSC-Derived CM Maturation

While intercellular interactions play crucial roles in cardiogenesis, providing these signals during differentiation and subsequent culture of hPSC-derived CMs will likely be insufficient to fully mature the CMs. Other microenvironmental cues, including mechanical forces, electrical stimulation, and ECM composition and mechanical properties also regulate CM phenotypes. Here we will discuss how these cues impact hPSC-derived CM phenotypes and how they can integrate into a CM manufacturing process. These cues and their effects on specific CM maturation phenotypes are shown in Table 2.

The contractile forces generated by the heart are necessary for cardiac homeostasis and impact heart development. To investigate the role of stresses on hPSC-derived CMs, Tulloch et al. assessed the effects of cyclic and static stresses on these cells (92). The cells were cast into a gel which was attached to a flexible silicon surface. Mesh tabs were used to introduce static stress whereas the deformable silicon substrate was stretched to induce cyclic stresses. Both cyclic and static stresses induced sarcomere organization, CM enlargement and alignment, and increased expression of *MYH7, CACNA1C, RYR2*, and *ATP2A2* (92). Cyclic stretch on the CMs cultured with HUVECs did not further enhance maturation in comparison to the monoculture though the cocultured CMs demonstrated increased DNA synthesis (92). By using CPCs to co-differentiate SMCs, ECs, and CMs together, Ruan et al. tested the effects of cyclic stretching on the resulting cardiac tissue constructs. Cyclic stretch increased the tissue stiffness and, in the hPSC-derived CMs, expression of cTnT, ratio of β-MHC:α-MHC, and cell contractility (86). Alternatively, Mihic et al. incorporated hESC-derived CMs into a gelatin sponge which could then be physically stretched and saw increased expression of the proteins Cx43 and MLC-2v and the genes *CACNA1C, SCN5A, KCNJ2, KCNH2, MYH7*, and faster Ca^2+^ handling (93). Incorporation of mechanical stresses into scalable CM manufacturing processes will likely prove challenging, although these cues may be effective when applied to cardiac tissues and might not be necessary during the CM differentiation phase of manufacturing.

Chan et al. employed electrical conditioning to simulate the cardiac conduction system signaling that developing myocytes are exposed to in the embryo in an effort to mature hESC-derived CMs (94). Electrically paced CMs demonstrated increased spontaneous and caffeine-induced calcium flux and upregulated expression of cardiac genes including *SCN5A, ATP2A2*, and *KCNH2*, suggesting enhanced electrophysiological changes in ion channel expression. Eng et al. further demonstrated the ability of electrical conditioning to enhance CM expression of cTnI and Cx43, and increase the fraction of rapidly depolarizing cells through inducing expression of *KCNH2*, a gene that encodes a potassium channel responsible for the ability of hPSC-derived CMs to adapt their autonomous beating rate to the rate of the stimulation (95). The ability to respond to signaling provided by the conduction system rather than to follow intrinsic pacing may reduce the risk of arrhythmias after cells are implanted.

The composition and mechanical properties of the ECM and cell microenvironment impacts hPSC differentiation and cell phenotypes (96). Decellularized tissues provide 3D scaffolds with the composition and structure of native ECM. Fong et al. cultured iPSC-derived CMs in decellularized fetal and adult bovine hearts in 3D culture (97). The decellularized adult heart ECM was found to be 10-fold stiffer than the decellularized fetal hearts and resulted in more extensive CM maturation, with increased expression of *JCN, CACNA1C, GJA1*, and *CASQ2*, compared to the CMs in decellularized fetal hearts. Herron et al. found that plating iPSC-derived CMs on soft PDMS gels increased cell size, Cx43, and cTnI expression, and CM contractility compared to CMs plated on glass (98). Culture on PDMS with an elastic modulus similar to that of cardiac tissue led to greater activation of β1 integrin receptors than culture on glass. When either the β1 integrin was directly inhibited by a neutralizing antibody or its downstream target, focal adhesion kinase, was inhibited, the CMs demonstrated a decrease in cTnI expression and cell size. This study further suggests the benefits of imitating both the composition of cardiac ECM and the stiffness of native heart tissue to accelerate CM maturation. Similarly, alignment of the ECM components also affects CM maturation. Li et al. cultured iPSC-derived CMs on electrospun, aligned nanofibers. CMs cultured on aligned fibers exhibited enhanced alignment, increased expression of MLC-2v and β-MHC, and higher electrical field potentials than CMs on random fibers and flat substrates (99). This highlights the ability of substrate topography to regulate both CM organization and maturation. Thus, one must consider ECM mechanics and organization as well as composition in designing a matrix for manufacturing CMs.

## Current Methods to Scale up hPSC-Derived CM Manufacturing

Many recent advances have been made toward up-scaling the production of hPSC-derived CMs. From optimizing the differentiation and identifying how to adjust crucial parameters during the process, the industry is getting closer to being able to reliably produce CMs on a large-scale basis. For example, Tohyama et al. recently demonstrated the ability to differentiate hPSC-derived CMs in monolayer culture in 10-layer, 1.2-L culture flasks with active gas ventilation, creating near a therapeutically relevant number of 1.5–2.8 × 10^9^ cells with >66% purity (100).

To reduce the cost of manufacturing, 3D suspension differentiation platforms have been developed. Suspension systems generate higher cell concentrations, reducing the cost of culture medium and the size of reactor needed. Ting et al. utilized microcarriers to transition from hESC expansion and differentiation to CMs on a flat 2D substrate to CM production in suspension (101). Microcarriers have a large surface area per volume and can be coated with different ECMs to facilitate cell attachment, proliferation, and differentiation. With gentle rocking during the stem cell culture and intermittent agitation during the differentiation, they obtained approximately 60% CM purity and about 200 million cells per 15-mL batch. With further development, microcarriers could provide a reliable and inexpensive method to produce clinically relevant numbers of hPSC-derived CMs. However, the resultant cells would likely have to be separated from the microcarriers prior to clinical use.

Recent advances also have demonstrated the ability to produce hPSC-derived CMs in suspension without microcarriers. For example, Nguyen et al. followed either the Laflamme et al. or Lian et al. directed differentiation protocols to generate CMs, singularized the CMs and plated them in microwells to form 3D aggregates before transferring the cells into a rotary orbital suspension culture (18, 21, 102). By optimization of the cell density in the microwells, they achieved almost 100% α-actinin^+^ cells in 3D culture. Both Chen et al. and Kempf et al. seeded undifferentiated hPSC aggregates in reactors to scale up production of hPSC-derived CMs in suspension culture (103, 104). Kempf et al. differentiated the hPSCs in a 100 mL stirred tank reactor, generating 40–50 million CMs per batch (104). Chen et al. produced 1.5–2 billion CMs in a 1 L spinner flask (103). To date, these suspension differentiation platforms have strived to produce pure populations of CMs. Moving forward, to introduce intercellular interactions in suspension CM manufacturing processes, direct cocultures may be achieved either through co-differentiation or introduction of other cell types during the differentiation. Furthermore, perfusion of media from a reactor containing other cell types could provide a method to introduce conditioned media to simulate coculture conditions. Alternatively an indirect coculture could be achieved through separation of the cell types with a membrane. The use of small molecules and growth factors to mimic intercellular interactions would provide a simpler, easier to scale, and likely more robust and cost-effective alternative to coculture platforms.

To create cardiac patches with mature hPSC-derived CMs, several studies have devised methods to culture the constituent cells on a large scale after differentiation. Shadrin et al. developed a method to create cardiospheres, using differentiated and singularized hPSC-derived CMs and culturing them in a hydrogel plug free-floating in medium. After 3 weeks in culture, the CMs demonstrated increased maturation with highly structured sarcomeres and T-tubules (105). The hydrogels were 36 mm × 36 mm, a size relevant for clinical application (105). In addition to allowing CM maturation, this method of culturing the CMs in the hydrogels post-differentiation is amenable to both coculture and scale-up. The introduction of other cell types could be easily achieved when encapsulating the cells into the hydrogel. Specific ECM components also could be incorporated into the hydrogel.

Biophysical techniques to maturate hPSC-derived CMs may prove difficult to integrate into a large-scale manufacturing process. Lux et al. created a bioreactor that can both provide cyclic mechanical stretch and perfusion of medium to cardiac patches up to 2.5 cm × 4.5 cm in size (106). Tandon et al. developed a portable bioreactor which can both provide perfusion and electrical stimulation to cardiac patches (107). Further engineering is required to scale-up these types of reactors, to design systems able to transmit electrical and mechanical cues in suspension. A comparison of the methods to scale-up production of mature hPSC-derived CMs is provided in Table 3.

**Table 3:** Comparison of scaling methods for the generation of mature human pluripotent stem cell (hPSC)-derived CMs.

Scaling method	Bioreactor capacity					Ease of potential incorporation						
	Size	Scalability	Purity	Cell yield	Starting cell type in bioreactor	Mechanical	Electrical	Perfusion	Membrane separated coculture	Extracellular matrix	Coculture of differentiated cells	Co-differentiation
10-layer tissue culture flasks (100)	1.2 L	HIGH	>66%	1.5–2.8B	hPSC	−	−	+	+	+	+	+
Microcarriers (101)	15 mL	HIGH	60%	0.2B	hPSC	−	−	+	+	+	+	+
3D cell aggregates (103)	1 L	HIGH	>90%	1.5–2B	hPSC	−	−	+	+	−	+	+
Cardiospheres (105)	Proof-of-concept	HIGH	Pre-purified	N/A	CMs	−	−	+	+	+	+	−
Perfusable, mechanical stimulation bioreactor (106)	N/A	LOW	Pre-purified	0.008B	CMs	+	−	+	−	+	+	−
Portable bioreactor (107)	N/A	LOW	Pre-purified	0.1B	CMs	−	+	+	−	+	+	−

*+ symbolizes minimal engineering to incorporate the maturation method into the bioreactor; − symbolizes significant engineering necessary*.

Research has begun to look at monitoring and controlling the cells during production to ensure the quality of the cell product. Kempf et al. investigated the effects of cell density and CHIR concentration on CM yield and purity (108). They found that the CHIR concentration needed to induce CM differentiation correlated with cell density. This suggests that CHIR concentration can be modified to account for differences in growth rates between different cell lines or different batches (109). Metabolic analysis of the media would also allow monitoring of the differentiation and maturation processes. For example, an increase in glycerophosphocholine and the glycerophosphocholine:phosphocholine ratio during maturation may be markers for the maturation state of the hPSC-derived CMs (66).

## Scalable Purification of hPSC-Derived CMs

After differentiation, hPSC-derived CMs will likely need to undergo a purification process to remove any traces of undifferentiated hPSCs or undesired differentiated cell types, and ensure a consistent product. Antibody-based purification methods are highly selective but costly to scale. Toward a negative selection process to remove undifferentiated hPSCs, Choo et al. developed an antibody, mAB 84, which selectively caused undifferentiated hESCs to die, likely through oncosis (110). This antibody could reduce the tumorigenic potential of cells differentiated from hPSCs, although this has not yet been shown to be a significant problem in preclinical models of hPSC-derived cardiac cell therapies. CM-specific surface markers allow separation of hPSC-derived CMs by MACS and FACs. MACS against SIRPA and VCAM1 has been used to yield >95% pure CMs (111, 112). However, there is a loss of CM yield following MACS (111). FACS also separates living cells based on expression of specific surface proteins. While it is highly efficient in terms of purity and yield, FACS is costly to scale. To eliminate the necessity of antibodies, Hattori et al. discovered that tetramethylrhodamine methyl ester perchlorate, a fluorescent dye that labels mitochondria, could be used to enrich hPSC-derived CMs to 99% purity (113). To enable their high energy utilization rate, CMs contains a large number of mitochondria in comparison to other cell types, with mitochondria comprising 30% of the CM volume (114).

Alternatively, genetic modification could allow purification of hPSC-derived CMs and other cardiac cells. Antibiotic resistance genes that are under the control of a cardiac-specific transcriptional regulator enables purification *via* negative antibiotic selection. For example, a 99% pure population of CMs differentiated from a murine stem cell line expressing aminoglycoside phosphotransferase under control of the *Myh6* promoter was isolated following treatment with G418 (115). Additionally, lineage-specific expression of fluorescent markers would allow FACS without the need for antibodies. Expressing eGFP from the *MYL2* promoter allowed purification of a 95% pure hESC-derived ventricular CM population (116). Miki et al. developed microRNA switches that can selectively terminate undesired cell types (117). Upon successful transfection of a specific microRNA switch corresponding to the desired cell type, the switch will induce apoptosis of all cell types except the target. By using microRNA 208a, they were able to enrich iPSC-derived CMs to a 95% purity with a loss of only 10% of the CMs (117). They further demonstrated the ability to use one switch with two targets, microRNA 208a for CMs and 126-3p for ECs, yielding a purified coculture of these two cell types (117). This method may be able to eliminate any non-CMs in tissue patches without disrupting the cellular organization. The main limitation of the microRNA switches and genetic selection methods is the necessity for either transient or permanent genetic modification of these cells, which will have to be thoroughly analyzed to establish safety *in vivo* before their potential use in regenerative medicine.

Finally, a metabolic selection may be used to purify CMs, taking advantage of their ability to use carbon sources that other cells cannot, such as lactate. Tohyama et al. demonstrated that lactate-containing medium can be used to generate 99% pure iPSC-derived CMs (118). This selection method was optimized in concert with differentiation of hPSC-derived CMs such that a pure population of cells was obtained within 20 days after the initiation of differentiation (24). By using a glucose-free medium, a pure CM population can be manufactured in a simple, defined, scalable process.

An overall comparison of the purification methods can be found in Table 4. After CM purification, the cells could undergo either density gradient or membrane filtration purification to remove any debris from the culture. Both of these methods are conducive to sterile large-scale cell manufacturing (119).

**Table 4:** Purification methods for large-scale production of human pluripotent stem cell (hPSC)-derived CMs.

	Scalability	Cost	Singularization required	Purity	Multiplexibility
Fluorescently activated cell sorting-mitochondria dyes (113)	Low	Low	Yes	99%	No
Fluorescently activated cell sorting-eGFP expression (116)	Low	Low	Yes	95%	Yes
Magnetically activated cell sorting (111, 112)	Medium	High	Yes	95%	Yes
Metabolic selection (24, 118)	High	Low	No	99%	No
Antibiotic selection (115)	High	Low	No	99%	Yes
Antibody-based negative selection for hPSCs (110)	High	Medium	No	98% removal of hPSCs	Yes
MicroRNA switches (117)	High	Medium	No	95%	Yes

## Preservation of hPSC-Derived CMs

Preservation would simplify the supply chain for meeting the clinical demand of hPSC-derived CMs. Typically the cells will be singularized then cryopreserved in a medium that contains a cryoprotectant, such as DMSO, and apoptosis inhibitors. When using the proprietary DMSO-containing cryopreservation solution CryoStor, Xu et al. found that the hESC-derived CMs had a recovery rate of 70–77% with similar viability and purity as before freezing (120). To enhance survival, the cells were pretreated with a pro-survival cocktail containing apoptosis inhibitors, K^+^ channel modulators, and growth factors for 24 h prior to cryopreservation (18). Following implantation into an ischemic rat heart, there were no differences in the sizes of grafts composed of hESC-derived CMs that had or had not undergone cryopreservation (120). Chong et al. also found no effect of hESC-derived CM cryopreservation on graft size after implantation of into mice hearts following myocardial infarction (13). DMSO causes cell toxicity and adverse reaction of patients, and thus must be removed from the cells prior to transplantation. DMSO alternatives including trehalose and poly-l-lysine, have been investigated although none have yet proven to effectively replace DMSO in cryopreservation media (121).

Alternatives to cryopreservation have been designed to simplify stabilization of hPSC-derived CMs. Correia et al. found that in 3D aggregates, about 70% of hPSC-derived CMs survived after storage at 4°C for up to 7 days (122). Although large scale manufacturing will likely require long-term cryopreservation, hypothermic stabilization may be suitable for transporting cells from a central manufacturing site to the clinic.

## Conclusion

Significant advances have recently been made in manufacturing relatively pure populations of CMs from hPSCs in fully defined processes, making the use hPSC-derived CMs for heart repair more plausible. The focus of research in this field is shifting to imparting more mature phenotypes in these cells to increase their safety and efficacy following transplantation. Additionally standards need to be defined to both quantify the extent of maturation and determine the level of maturation that is optimal for transplantation. The ratio of cTnI:ssTnI expression was proposed to be such a marker, but it is not yet clear how to assess electrical, mechanical, or metabolic maturation.

Recent efforts to simulate the intercellular interactions found in the heart *in vivo* during hPSC differentiation to CMs *in vitro* have demonstrated the importance of incorporating ECM, juxtacrine, and paracrine interactions between CMs, ECs, and fibroblasts. Of these, fibroblast ECM and EC juxtacrine signaling have been shown to enhance maturation phenotypes in hPSC-derived CMs. In addition, several experiments have pointed toward the necessity to use cardiac-specific cell types to induce maturation with the coculture. This should be further investigated to reveal mechanisms by which fibroblasts and ECs induce specific phenotypes in CMs. These cues could then be engineered into CM manufacturing processes in simpler manner than coculture. Also, efforts to discover genetic and epigenetic regulators of cell state, growth factors, hormones, and metabolites that enhance maturation would facilitate scalable production of hPSC-derived CMs.

Introduction of cardiac intercellular interactions *via* either microtissues or co-differentiation has been shown to enhance CM survival and engraftment *in vivo* in addition to CM maturation. Thus far the potential to co-differentiate cardiac cells from stem and progenitor cell types has not been investigated in sufficient depth due to insufficient control of these complex differentiation systems. In addition, co-differentiation would likely require purification using methods such as microRNA switches or antibiotic or metabolic selection. The potential of CPCs to form appropriately structured myocardial tissue is a powerful advantage in developing cardiac regenerative therapies and should be investigated more extensively.

Mechanical and electrical simulation are effective means to accelerate maturation in hPSC-derived CMs but are difficult to incorporate in scalable manufacturing processes. Design of bioreactors to deliver these biophysical cues will likely improve CM and cardiac tissue manufacturing processes. A better mechanistic understanding of mechanotransduction during differentiation and maturation would enable alternative biochemical or genetic strategies to modulate these pathways during CM manufacturing. Control of ECM organization, stiffness, and structure represents another promising approach to regulate hPSC-derived CM maturation.

To date, no single method has proved effective in inducing maturation in hPSC-derived CMs. A combination of factors will likely be necessary to generate CMs of the appropriate maturity for regenerative therapies. Identification of effective strategies will be enabled by studies that relate the effects of maturation cues on specific phenotypes and identify mechanisms by which these signals impart maturation.

## Author Contributions

KD and SP wrote the manuscript and read and approved the submitted version.

## Conflict of Interest Statement

The authors declare that the research was conducted in the absence of any commercial or financial relationships that could be construed as a potential conflict of interest.
